# Fusion Protein of Rotavirus VP6 and SARS-CoV-2 Receptor Binding Domain Induces T Cell Responses

**DOI:** 10.3390/vaccines9070733

**Published:** 2021-07-02

**Authors:** Kirsi Tamminen, Suvi Heinimäki, Stina Gröhn, Vesna Blazevic

**Affiliations:** Vaccine Development and Immunology/Vaccine Research Center, Faculty of Medicine and Health Technology, Tampere University, Arvo Ylpön katu 34, FI-33520 Tampere, Finland; suvi.heinimaki@tuni.fi (S.H.); stina.grohn@tuni.fi (S.G.)

**Keywords:** SARS-CoV-2, COVID-19, delivery platform, vaccines, VP6, T cells, antibodies, immune response

## Abstract

Vaccines based on mRNA and viral vectors are currently used in the frontline to combat the ongoing pandemic caused by the novel Severe Acute Respiratory Syndrome Coronavirus-2 (SARS-CoV-2). However, there is still an urgent need for alternative vaccine technologies inducing/boosting long-lasting and cross-reactive immunity in different populations. As a possible vaccine candidate, we employed the rotavirus VP6-protein platform to construct a fusion protein (FP) displaying receptor-binding domain (RBD) of SARS-CoV-2 spike protein (S) at the N-terminus of VP6. The recombinant baculovirus-insect cell produced VP6-RBD FP was proven antigenic in vitro and bound to the human angiotensin-converting enzyme 2 (hACE2) receptor. The FP was used to immunize BALB/c mice, and humoral- and T cell-mediated immune responses were investigated. SARS-CoV-2 RBD-specific T cells were induced at a high quantity; however, no RBD or S-specific antibodies were detected. The results suggest that conformational B cell epitopes might be buried inside the VP6, while RBD-specific T cell epitopes are available for T cell recognition after the processing and presentation of FP by the antigen-presenting cells. Further immunogenicity studies are needed to confirm these findings and to assess whether, under different experimental conditions, the VP6 platform may present SARS-CoV-2 antigens to B cells as well.

## 1. Introduction

Since the recent emergence of Severe Acute Respiratory Syndrome Coronavirus-2 (SARS-CoV-2) in December 2019 causing the COVID-19 disease and pandemic [1], there have already been several vaccines approved for human use, and numerous vaccine candidates are in the different phases of clinical trials or under early development [2]. Most of the vaccine candidates against SARS-CoV-2 target the spike (S) protein, especially its receptor binding domain (RBD) responsible for cell attachment, entry, and, therefore, infection [3]. The neutralizing (Ne) antibodies against SARS-CoV-2 RBD are considered the main correlates of protection from infection and disease [4]. All vaccines currently used to mass immunize the human population, including mRNA-based BNT162 (Pfizer/BioNTech, Mainz, Germany) and mRNA-1273 (Moderna, Inc., Cambridge, MA, USA) or adenovirus vector-based AZD1222 (Oxford/AstraZeneca, Cambridge, UK), induce high levels of Ne antibodies up to several months after the vaccination [5]. However, it is still unknown whether long-lasting and cross-protective immunity against newly emerging SARS-CoV-2 variants of concern [6] will be generated with the currently available vaccines. Therefore, a better understanding and characterization of the immune responses against SARS-CoV-2 and the role of T cell immunity in protection is needed. In different viral infections, including influenza virus [7,8], T cell immunity plays a crucial role in memory B cell response generation and cross-protective and long-lasting immunity. There is increasing evidence that cellular immunity, namely SARS-CoV-2-specific CD8^+^ cytotoxic T cells and/or Th1-biased CD4^+^ helper T cells, also plays an important role in long-term protection against SARS-CoV-2 [9]. It is highly possible that different types of vaccine technologies or/and additional, more conserved SARS-CoV-2 antigens will be needed to boost and broaden the immune response after initial priming with the current vaccines. We and others have previously stated that the recombinant rotavirus (RV) VP6 protein, produced by the baculovirus (BV) insect cells protein expression system, in addition to being a nonreplicating subunit RV vaccine candidate [10,11,12,13], possesses self-adjuvating abilities and acts as a delivery vehicle for particulate antigens [14,15]. VP6 oligomeric nanostructures, such as nanospheres or nanotubules, are optimal-sized particles (~100–1000 nm) to be uptaken by antigen-presenting cells (APCs) [16,17], thus efficiently activating adaptive immune responses. High immunogenicity and the intrinsic immunostimulatory effect of VP6 have been further harnessed for the use of VP6 as a platform for heterologous antigen presentation [18,19,20]. Recently, we showed [20] that VP6 easily accommodates foreign antigen sequences (universal influenza virus antigens) from ca. 20 to 160 amino acids (aa) at different insertion sites within the surface loops and both termini, making it an excellent delivery platform for different vaccine antigens, including SARS-CoV-2. In the present study, RV VP6 was employed as a platform to deliver and display the SARS-CoV-2 RBD antigen at the N-terminus (N-t). The constructed fusion protein (FP) was used to immunize BALB/c mice, and RBD-specific antibodies and T cell responses were investigated. This type of a recombinant subunit protein vaccine exhibits a high safety profile and could be used as a standalone vaccine or as a heterologous boost to different priming strategies with mRNA, DNA, or virus vector-based vaccines.

## 2. Materials and Methods

### 2.1. Cloning and Production of VP6-RBD Fusion Protein

A recombinant VP6-RBD FP was designed by inserting SARS-CoV-2 RBD (aa 319–541, GenBank ID QHD43416.1, PANGO lineage B [21]) at N-t of human RV VP6 (1–397 aa, GenBank ID GQ477131) by genetic fusion as previously described [20]. A flexible linker sequence ([G_4_S]_3_) was inserted between RBD and the VP6 backbone to sustain the correct folding of both domains. The FP DNA sequence was synthetized and cloned into pFastBac™1 vector by GeneArt (Thermo Fisher Scientific, Waltham, MA, USA) and further used to generate recombinant bacmids by the Bac-to-Bac^®^ Baculovirus Expression system (Invitrogen, Carlsbad, CA, USA). The recombinant bacmids were used to transfect *Spodoptera frugiperda* (Sf9) insect cells to generate baculovirus stock, which was amplified as earlier described [10,20]. Sf9 cells were infected with high titer baculovirus stock at a density of 1 × 10^6^ cells/mL and multiplicity of infection (MOI) of 1 and 6 days postinfection. Cell pellet was collected by centrifugation (1000× *g*, 20 min, +4 °C).

### 2.2. Extraction and Crude Purification of VP6-RBD Fusion Protein

To extract VP6-RBD FP from the cell pellet, a method described by O’Shaughnessy and Doyle [22] was exploited. Briefly, pellet was disrupted by lysis buffer (25 mM Tris–HCl, 10 mM NaCl, 5 mM MgCl_2_, pH 7.5) in the presence of protease inhibitors (PMSF and leupeptin) and 10% nonyl phenoxypolyethoxylethanol (NP-40, all from Sigma-Aldrich, St. Louis, MO, USA). After centrifugation (10,000× *g*, 15 min), 5% sodium deoxycholate (Sigma-Aldrich) was added to disrupt nuclei, releasing DNA, which was digested o/n (+4 °C) with DNase I (Thermo Fisher). The pellet was treated with two-step addition of solubilization buffers with an increasing urea concentration (25 mM Tris–HCl, 1 mM EDTA containing 2 or 8 M urea). As the FP still predominately remained insoluble in the pellet (Western blot analysis, data not shown), the pellet was dissolved in 6 M guanidine–HCl supplied with 20 mM dithiothreitol. Guanidine was removed by dialysis (20K MWCO Slide-A-Lyzer™ cassette, Thermo Fisher) against 6 and 3 M urea in 50 mM Tris-HCl and subsequently against phosphate buffered saline (PBS). After centrifugation (10 min, 4000× *g*), the pellet was resuspended in 8 M urea in 25 mM Tris-HCl, following the refolding of FPs by sequential dialysis against a slowly decreasing urea concentration (5–0 M urea in 50 mM Tris-HCl, pH 8). Finally, the soluble protein preparation was clarified by centrifugation (10,000× *g*, 10 min) to separate the supernatant, containing soluble FP, from insoluble material remaining in the pellet.

### 2.3. RV VP6, SARS-CoV-2 Proteins, and Synthetic Peptides

Recombinant human RV VP6 protein, used as a control antigen for analytical in vitro assays, was produced by our laboratory as previously described [10,23]. Active SARS-CoV-2 trimeric S protein in the prefusion conformation (CoV-2 S, R&D Systems, Minneapolis, MN, USA) was used as a standard SARS-CoV-2 vaccine antigen to immunize mice [24] and as an antigen in analytical assays. Recombinant SARS-CoV-2 RBD (CoV-2 RBD, Sanyou Biopharmaceuticals Co., Ltd., Shanghai, China) and nucleoprotein (CoV-2 NP, Sanyou Biopharmaceuticals) were used as antigens for the analytical methods only. PepTivator SARS-CoV-2 S1 (15-mer sequences with 11 aa overlap, covering the N-terminal S1 domain, aa 1-692) and NP (15-mer sequences with 11 aa overlap, covering the complete sequence of NP) peptide pools were obtained from Miltenyi Biotec (Lund, Sweden).

### 2.4. VP6-RBD Fusion Protein Characterization and Receptor Binding

The expression and purity of VP6-RBD FP were characterized with SDS-PAGE using Mini Protean TGX Precast gels (Bio-Rad Laboratories, Hercules, CA, USA) and PageBlue Protein Staining Solution (Thermo Fisher). Total protein concentration was determined with a Pierce BCA kit (Thermo Fisher) following densitometric analysis with a ChemiDoC XRS Imager (Bio-Rad) to determine FP quantity in the soluble protein preparation. The FP identity and antigenicity was confirmed by Western blot as previously described [20] using rabbit anti-RV polyclonal antibody (1:500, Antibodies-online GmbH, Aachen, Germany) and rabbit anti-SARS-CoV-2 S1 antibody (1:200, Invitrogen), following 1:5000 diluted anti-rabbit HRP-conjugated antibody (Abcam, Cambridge, UK). The binding of VP6-RBD FP, trimeric CoV-2 S (a positive control), and CoV-2 NP (a negative control) to human angiotensin-converting enzyme 2 (hACE2) receptor was performed as earlier described [25] with slight modifications. hACE2 (aa 18-740), hFc Tag Recombinant Protein (Invitrogen) was immobilized on a 96-well polystyrene plate (Corning, New York, NY, USA) at 1 µg/mL in PBS (o/n, +4 °C). VP6-RBD, CoV-2 S, and NP proteins were added with increasing concentration (0–240 ng/well) on the plate. After washing the plate, hACE2-bound proteins were detected with rabbit anti-SARS-CoV-2 S1 antibody (Invitrogen) and anti-rabbit IgG-HRP. OPD substrate (Sigma-Aldrich) was added for 30 min, and after stopping the reaction with 2 M H_2_SO_4_, optical density at 490 nm (OD_490_) was measured using a microplate reader (Victor^2^, Perkin Elmer, Waltham, MA, USA). A background signal, obtained from hACE2-coated wells incubated with the sample buffer only, was subtracted from all OD readings at a plate.

### 2.5. Mice Immunization

Three groups of six-week-old female BALB/c mice (Envigo, Horst, The Netherlands) were immunized subcutaneously two times (on study week 0 and 3) with 55 µg VP6-RBD (6 mice/group), 10 µg active trimer CoV-2 S (R&D Systems, 4 mice/group, a positive control group), or carrier (PBS) only (4 mice/group, a negative control group). VP6-RBD was adjuvanted with aluminum hydroxide (100 µg/dose), and two of the positive control mice received CoV-2 S adjuvanted with alum (100 µg/dose), while two received CoV-2 S in PBS only. Mice were sacrificed on study week 5, and whole blood (serum) and splenocytes were collected and frozen as previously described [26]. All procedures were authorized and conducted under the guidelines of the Finnish National Animal Experiment Board (permission number ESAVI/10800/04.10.07/2016).

### 2.6. SARS-CoV-2 and RV VP6-Specific Serological Immunoassays

RV VP6 and SARS-CoV-2-specific antibodies in immunized mice sera were measured by enzyme-linked immunosorbent assay (ELISA) [26]. RV VP6 (2 µg/mL in PBS), CoV-2 S, RBD, or NP (each 1 µg/mL in PBS) were coated on 96-well ELISA-plates (Corning) o/n at +4 °C. Mice sera were diluted twofold from 1:100 and added to the plates. The bound antibodies were detected by 1:4000 diluted antimouse IgG-HRP (Sigma-Aldrich) and OPD substrate as described above. Negative control mouse sera were used to determine the cutoff value (mean OD_490_ + 3 × SD). Samples with a net OD_490_ above the set cutoff value and >0.1 OD were considered positive. The end-point antibody titers were defined as a reciprocal of the highest sample dilution with an OD_490_ above the set cutoff value. If the starting dilution resulted in an OD value below the set cutoff, the sample was given an arbitrary value half of the starting dilution. Sera of mice immunized with VP6-RBD FP were pooled (1:100 dilution) to detect CoV-2 RBD, CoV-2 S, and VP6 in a Western blot as previously described [20].

### 2.7. ELISPOT-IFN-γ

An enzyme-linked immunospot assay (ELISPOT) was used to quantify IFN-γ production from VP6-RBD FP, CoV-2 S, or mock (PBS) immunized mice splenocytes in response to SARS-CoV-2 S1 (covering the RBD domain) and SARS-CoV-2 NP-derived synthetic peptide pools as previously described for other peptides [26]. Briefly, 96-well MultiScreen HTS-IP filter plates (Millipore, Billerica, MA, USA) were coated with antimouse IFN-γ (Mabtech Ab, Nacka Strand, Sweden). The S1 peptide pool (0.2 and 1 µg/mL), NP pool (1 µg/mL, a negative control), or Concanavalin A (ConA, Sigma-Aldrich, 10 µg/mL), used as a positive viability control, were added to stimulate individual mice splenocytes (0.2 × 10^6^ cells/well). Cell culture medium (CM) was used as a background control. The plates were incubated for 20 h at 37 °C and 5% CO_2_, following spot development as previously described [26]. The plates were analyzed using an ImmunoSpot^®^ automatic CTL analyzer (CTL-Europe GmbH, Bonn, Germany). The results are expressed as mean spot forming cells (SFCs)/10^6^ live splenocytes. A sample was considered positive if the quantity of SFC/10^6^ cells was above the maximum background level (cutoff value) calculated from CM wells (mean SFC/10^6^ cells + 3 × SD).

### 2.8. Statistics

A Mann–Whitney *U*-test was used to compare differences in nonparametric observations between two independent groups. Statistical significance was defined as *p* < 0.05, and all hypothesis testing was two tailed. Statistical analyses were conducted using GraphPad Prism (San Diego, CA, USA) version 8.3.0.

## 3. Results

### 3.1. Production and Characterization of the Crude Purified VP6-RBD Fusion Protein

**Figure 1:**
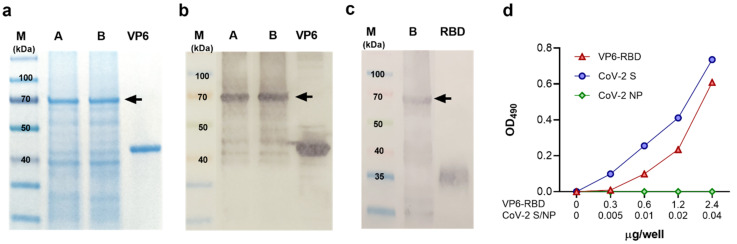
Characterization of VP6-RBD fusion protein. Rotavirus VP6 protein was used as a backbone to generate fusion protein (FP) carrying SARS-CoV-2 Spike (S) protein derived receptor-binding domain (RBD) as described in the Materials and Methods. The FP was crude purified from Sf9 insect cell pellets after 6-day culture with recombinant baculovirus and separated (5 μg/lane of total protein) under reducing conditions in SDS-PAGE following staining with Coomassie blue (**a**) or immunoblotting using primary antibodies against VP6 (**b**) or SARS-CoV-2 S protein (**c**). VP6-RBD FP (~70 kDa) before (A) and after (B) final clarification step is indicated by an arrow (**a**–**c**). Wild-type VP6 (~42 kDa) and commercial RBD protein (~23 kDa, migrating as a higher molecular weight due to glycosylation) were used as positive controls in SDS-PAGE and/or Western blot. Molecular weight marker (M) is shown with the respective molecular sizes (kDa). Binding of VP6-RBD, SARS-CoV-2 S (CoV-2 S, a positive control), and SARS-CoV-2 nucleoprotein (CoV-2 NP, a negative control) to immobilized human angiotensin-converting enzyme 2 (hACE2) by an ELISA-based binding assay (**d**). Optical density (OD) values at 490 nm of VP6-RBD (0–2.4 µg/well), CoV-2 S, and NP (both 0–0.04 µg/well) binding to 0.05 µg/well hACE2 are shown.

In preliminary experiments, the insect-cell-produced VP6-RBD was found intracellularly located and insoluble (data not shown). Therefore, cell pellets were subjected to pellet extraction and protein solubilization procedures as described in the Materials and Methods. The presence of VP6-RBD (theoretical size app. 70 kDa) after extraction/solubilization procedures was confirmed by SDS-PAGE (Figure 1a, lane A) and VP6-specific Western blot (Figure 1b, lane A) analysis. These analyses also showed that VP6-RBD remained in the supernatant (Figure 1a,b, lane B) after the final centrifugation step, indicating successful FP solubilization. The presence of SARS-CoV-2 RBD-insert was confirmed by SARS-CoV-2 S antibody staining in the Western blot (Figure 1c, lane B). Moreover, the immunoblotting experiments of VP6-RBD FP revealed that the majority of VP6 protein, as well as RBD, is in a conjugated form (Figure 1b,c). The total protein concentration of the final protein preparation was 1.37 mg/mL, and the percentual fraction of VP6-RBD FP was determined by densitometry analysis of the SDS-PAGE-separated proteins shown in Figure 1a (lane B) (data not shown). The theoretical FP concentration (calculated from the densitometry results, respectively) in the final preparation was 0.288 mg/mL, and the total FP yield was 2.5 mg/L. Functionality and structural integrity of the RBD-insert in the VP6-RBD FP was investigated using hACE2 receptor binding experiments. Recombinant CoV-2 S trimer was used as a positive and CoV-2 NP as a negative control in the assay. FP binding to hACE2 (Figure 1d) demonstrated that RBD is expressed and is functional. Approximately 60× more RBD in the FP was needed to obtain a binding profile to the hACE2 receptor similar to the spike protein (2.4 µg compared to 0.04 ug/well). The negative control CoV-2 NP did not bind to hACE-2 (Figure 1d).

### 3.2. SARS-CoV-2 and RV VP6-Specific Antibody Responses

**Figure 2:**
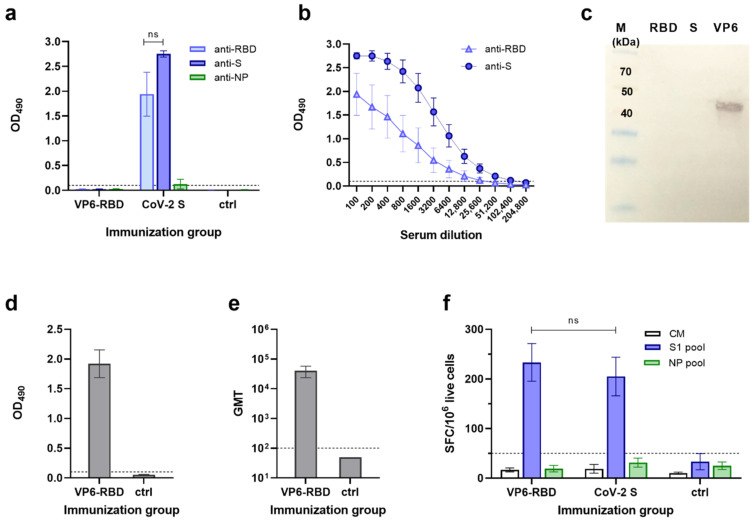
SARS-CoV-2 and VP6-specific antibody and T cell responses. Serum IgG antibody responses in mice following immunization with VP6-SARS-CoV-2 receptor-binding domain (VP6-RBD) fusion protein (FP), SARS-CoV-2 Spike (CoV-2 S, a positive control), or carrier only (PBS, a negative control) were analyzed by antigen-specific ELISAs and/or Western blot. SARS-CoV-2 S, RBD, and nucleoprotein (NP)-specific IgG antibodies are shown, represented by mean optical density (OD) values with standard error of the mean (SEM) of sera diluted 1:100 (**a**) and serum titration curves of CoV-2 S immunized mice (**b**). VP6-RBD FP immune sera were further used as a pool of individual sera (1:100 dilution) to detect SARS-CoV-2 RBD, S, and VP6 (each 1μg/lane) proteins under denaturing conditions in a Western blot (**c**). Molecular weight marker (M) is shown with the respective molecular sizes (kDa). VP6-specific IgG responses of VP6-RBD FP immunized and control mice are illustrated by mean OD values with SEM (**d**) and geometric mean titer (GMT) with 95% confidence intervals (**e**). If the starting dilution resulted in an OD value below the set cutoff, the sample was given an arbitrary value half of the starting dilution (ctrl, (**e**)). T cell responses of immunized and control mice splenocytes were analyzed by ELISPOT IFN-γ against SARS-CoV-2 S1 and NP (a negative control) peptide pool stimulation (**f**). Cell culture medium (CM) alone was used to determine background cytokine release. Mean IFN-γ spot-forming cells (SFC)/10^6^ live splenocytes of duplicate wells with SEM are shown. Dashed horizontal lines in the panels represent the cutoff value for ELISA ((**a**,**b**,**d**,**e**), mean OD_490_ + 3 × SD and at least 0.1 OD of negative control mice) and ELISPOT ((**f**), mean SFC/10^6^ cells + 3 × SD of CM wells). Statistical significance was defined as *p* < 0.05 and hypothesis testing was two tailed. ns: not significant *p*-value.

Individual serum samples of VP6-RBD FP and CoV-2 S immunized mice were tested for the presence of SARS-CoV-2-specific antibodies in ELISA against CoV-2 S and RBD recombinant proteins. VP6-RBD completely failed to induce a SARS-CoV-2-specific IgG response (Figure 2a). To the contrary, CoV-2 S, used as a positive immunization control antigen, induced a robust IgG response (Figure 2a,b) in each animal against CoV-2 S and RBD with GMT 86108 (95% CI 21,493–344,968) and 10763 (95% CI 1108–104,580), respectively. As comparable results were obtained in the positive control group of mice immunized with or without the adjuvant (data not shown), the results were combined. We next performed an immunoblotting experiment with denatured CoV-2 S and RBD (soluble monomer) to investigate if there were antibodies generated to nonconformational epitopes in VP6-RBD immunized mice. The results show that there were no antibodies detected in either of these proteins, only in the recombinant wild-type VP6 used as a control (Figure 2c). As pooled sera of six mice were used in the immunoblotting experiment, we determined the IgG antibody response of individual mice against the VP6 carrier protein in ELISA. A robust anti-VP6 response in VP6-RBD FP immunized mice (Figure 2d,e) with GMT 36204 (95% CI 19,699–66,537) was detected, confirming successful immunization. Negative control mice sera were negative for SARS-CoV-2 and VP6-specific antibodies (Figure 2a,d, respectively).

### 3.3. SARS-CoV-2-Specific T Cell Responses

To evaluate SARS-CoV-2-specific T cell responses in the VP6-RBD FP and CoV-2 S immunized mice, individual mice splenocytes were stimulated with SARS-CoV-2 S1 and NP peptide (negative control) pools, and IFN-γ production was measured by ELISPOT (Figure 2f). The VP6-RBD immunized mice splenocytes responded to the SARS-CoV-2 S1 peptide pool stimulation ex vivo by robust IFN-γ production (233 ± 38 SFC/10^6^ cells). A similar level of IFN-γ production (*p* = 0.919) was detected by the S1 peptide pool stimulated splenocytes from CoV-2 S immunized mice (205 ± 39 SFC/10^6^ cells). Importantly, there was no significant IFN-γ response detected against the negative control NP peptide pool in any of the mice immunized with VP6-RBD FP (19 ± 7 SFC/10^6^ cells) or CoV-2 S (32 ± 9 SFC/10^6^ cells). Mice immunized with PBS carrier only were negative for IFN-γ production to both peptide pools (Figure 2f). Cell viability, controlled by Con A stimulation, was similar in all groups (data not shown), and background IFN-γ production by splenocytes in CM only was < 40 SFC/10^6^ cells.

## 4. Discussion

Although several SARS-CoV-2 vaccines are approved for human use worldwide, many different vaccination approaches and vaccine candidates to, e.g., improve the longevity of the protective immune responses and to induce cross reactivity against emerging SARS-CoV-2 strains, are under development [2,24]. We have used the RV VP6 protein delivery platform carrying SARS-CoV-2 RBD antigen as a vaccine candidate and investigated its potential to induce RBD-specific antibodies and T cell responses. Of the several possible insertion sites within the VP6 sequence [18,19,20], we selected the N-t, based on recently published results [20], to avoid the steric hinderance on the VP6 structure by a large insert, and to sustain the ability of VP6 to trimerize [18]. This type of a subunit protein vaccine is extremely safe [27] and could be used as a standalone vaccine or as a heterologous boost to different vaccination priming strategies. In particular, viral vector-based vaccines, including different adenovirus vectors, may need a heterologous boost due to the vector-induced immune responses over time [28]. In addition, platform technology is very adaptable, enabling the rapid exchange of antigens of the continuously emerging SARS-CoV-2 variants. SARS-CoVs use the hACE2 receptor to enter and infect host cells [29]. Current vaccine strategies against SARS-CoV-2 primarily aim to induce the high-level Ne antibodies against the RBD of S protein to prevent the COVID-19 disease and infection. Although we used alum, a Th2 type adjuvant, in our immunization experiments with VP6-RBD FP, to our great surprise, no antibodies were detected against SARS-CoV-2 RBD or S protein. However, SARS-CoV-2 RBD-specific T cells were induced at a similar quantity to the full-length trimeric S-protein-induced responses. The reasons behind these observations are not defined and require further investigation. It is known that a low antigen dose may induce T cell responses in the absence of antibodies [30]. The 10-µg CoV-2 S protein dose, which induced antibodies specific for S and RBD proteins, contains approximately 1.75 µg RBD. Although there was ~20 µg SARS-CoV-2 RBD in the FP per dose used for immunizing mice, it is possible that antibody binding sites are buried by the VP6 protein structure while T cell epitopes are readily available after the FP uptake, processing, and presentation by APCs. Indeed, the hACE2 binding results showed that approximately 60× more RBD in the form of FP, compared to the S protein, is needed to obtain similar binding to the hACE2 receptor, indicating that binding sites in FP are at least partially masked by the VP6. In similar fashion, these conformational sites on RBD might not be available at a sufficient level for B cell receptor recognition and subsequent antibody generation in vivo. However, VP6-RBD FP antigenicity was demonstrated in immunoblotting experiments as the polyclonal antibody specific for SARS-CoV-2 S bound to denatured FP transferred on the blot. It is therefore possible that antibody priming, and generation in vivo, requires a larger quantity of RBD antigen in the form of FP than used under our experimental conditions. However, as these are novel and early observations, the results need to be confirmed by further experiments. Immunogenicity studies in mice using a wide dose range of VP6-RBD FP, different adjuvants (or a combination of these) and delivery methods, additional boosting immunizations to promote antibody induction, and an extension of the study schedule to investigate long-term immunity are warranted. It would be important to perform immunization experiments with our candidate vaccine in yet another animal model, such as in Syrian hamsters, which are susceptible to SARS-CoV-2 infection and are utilized to study virus transmission between hosts [31]. This model could be used to determine the protection from infection induced by the VP6-RBD vaccine candidate. Furthermore, VP6 platform technology fused with antigens derived from different emerging SARS-CoV-2 variants could be tested for protection as well. Yet another approach could employ VP6 surface loops to insert shorter stretch(es) from the RBD-enclosed receptor binding motif (RBM), containing functionally important epitopes for ACE2 interaction [29,32], as we have recently done with influenza virus antigens [20]. With this approach, only the key epitopes for Ne antibodies would be readily available for B cell recognition, which would reduce the risk of adverse antibody-dependent enhancement (ADE) caused by the induction of suboptimal non-neutralizing antibodies [33]. We also aim to determine which cell type [23] is responsible for IFN-γ production in response to the SARS-CoV-2 S1 domain-derived synthetic peptides that we detected. However, it is likely that both CD4^+^ and CD8^+^ T cells are generated by the VP6-RBD FP immunization and secrete IFN-γ ex vivo. Typically, CD4^+^ and CD8^+^ T cells recognize 12–15-aa- and 8–10-aa-long peptide sequences presented by the major histocompatibility complex (MHC) molecules on the surface of APC. The peptides contained in the S1 pool are 15 aa long and therefore contain putative epitopes for both cell types. In addition, we will investigate production of other cytokines, such as interleukin (IL)-4, IL-5, IL-10, and IL-17, in immunized animals. As Th2-type cytokines, especially IL-5, have been implicated in the induction of vaccine-associated disease enhancement [34,35], it is necessary to confirm that our SARS-CoV-2 vaccine candidate does not induce adverse immunity. An antigen delivery platform, such as the one described here, which would primarily induce T cell immunity in the absence of antibody generation, might be a valuable vaccination strategy against pathogens such as *Mycobacterium tuberculosis*, where T cell response (especially IFN-γ production by CD4^+^ T cells) is the only protective response needed [36]. In addition, such a vaccine platform carrying a foreign antigen could be used in a combination or as a boost for vaccines with a negligent or absent T cell response induction. The current SARS-CoV-2 vaccines approved for human use induce protection associated with high S/RBD-specific antibody levels, which lasts up to several months [5]. It is still not known whether long-lasting protective immunity will be generated with the current SARS-CoV-2 vaccines in use. Next-generation vaccine approaches will probably be needed to boost prior immunized people. Booster vaccinations inducing SARS-CoV-2 T cell immunity instead of Ne antibodies might be needed to prolong duration and to induce cross-protective responses against emerging SARS-CoV-2 variants of concern. It has been suggested that cross-reactive T cells induced by seasonal influenza vaccinations might mediate protection from pandemic influenza virus infection in the absence of Ne antibodies to hemagglutinin and neuraminidase [37]. T cell immunity may also play an important role in the protection from SARS-CoV-2 infection and the induction of long-lasting, cross-reactive protection, as is the case in other viral infections [7,37,38,39].

## Data Availability

The data presented in this study are available upon reasonable request.

## References

[1] Cucinotta D., Vanelli M. (2020). WHO declares COVID-19 a pandemic. Acta Biomed..

[2] COVID-19 Vaccine Tracker and Landscape. https://www.who.int/publications/m/item/draft-landscape-of-covid-19-candidate-vaccines.

[3] Dai L., Gao G.F. (2021). Viral targets for vaccines against COVID-19. Nat. Rev. Immunol..

[4] Addetia A., Crawford K.H., Dingens A., Zhu H., Roychoudhury P., Huang M.L., Jerome K.R., Bloom J.D., Greninger A.L. (2020). Neutralizing antibodies correlate with protection from SARS-CoV-2 in humans during a fishery vessel outbreak with high attack rate. J. Clin. Microbiol..

[5] Golob J.L., Lugogo N., Lauring A.S., Lok A.S. (2021). SARS-CoV-2 vaccines: A triumph of science and collaboration. JCI Insight.

[6] Garcia-Beltran W.F., Lam E.C., St Denis K., Nitido A.D., Garcia Z.H., Hauser B.M., Feldman J., Pavlovic M.N., Gregory D.J., Poznansky M.C. (2021). Multiple SARS-CoV-2 variants escape neutralization by vaccine-induced humoral immunity. Cell.

[7] Sridhar S., Begom S., Bermingham A., Hoschler K., Adamson W., Carman W., Bean T., Barclay W., Deeks J.J., Lalvani A. (2013). Cellular immune correlates of protection against symptomatic pandemic influenza. Nat. Med..

[8] Wilkinson T.M., Li C.K., Chui C.S., Huang A.K., Perkins M., Liebner J.C., Lambkin-Williams R., Gilbert A., Oxford J., Nicholas B. (2012). Preexisting influenza-specific CD4^+^ T cells correlate with disease protection against influenza challenge in humans. Nat. Med..

[9] Iqbal H. (2020). The importance of cell-mediated immunity in COVID-19—An opinion. Med. Hypotheses.

[10] Blazevic V., Lappalainen S., Nurminen K., Huhti L., Vesikari T. (2011). Norovirus VLPs and rotavirus VP6 protein as combined vaccine for childhood gastroenteritis. Vaccine.

[11] Lappalainen S., Pastor A.R., Tamminen K., Lopez-Guerrero V., Esquivel-Guadarrama F., Palomares L.A., Vesikari T., Blazevic V. (2014). Immune responses elicited against rotavirus middle layer protein VP6 inhibit viral replication in vitro and in vivo. Hum. Vaccines Immunother..

[12] Pastor A.R., Rodriguez-Limas W.A., Contreras M.A., Esquivel E., Esquivel-Guadarrama F., Ramirez O.T., Palomares L.A. (2014). The assembly conformation of rotavirus VP6 determines its protective efficacy against rotavirus challenge in mice. Vaccine.

[13] Caddy S.L., Vaysburd M., Wing M., Foss S., Andersen J.T., O’Connell K., Mayes K., Higginson K., Iturriza-Gomara M., Desselberger U. (2020). Intracellular neutralisation of rotavirus by VP6-specific IgG. PLoS Pathog..

[14] Malm M., Tamminen K., Lappalainen S., Vesikari T., Blazevic V. (2016). Rotavirus recombinant VP6 nanotubes act as an immunomodulator and delivery vehicle for norovirus virus-like particles. J. Immunol. Res..

[15] Tamminen K., Heinimaki S., Vesikari T., Blazevic V. (2020). Rotavirus VP6 adjuvant effect on norovirus GII.4 virus-like particle uptake and presentation by bone marrow-derived dendritic cells in vitro and in vivo. J. Immunol. Res..

[16] Rodriguez M., Wood C., Sanchez-Lopez R., Castro-Acosta R.M., Ramirez O.T., Palomares L.A. (2014). Understanding internalization of rotavirus VP6 nanotubes by cells: Towards a recombinant vaccine. Arch. Virol..

[17] Tamminen K., Heinimaki S., Grohn S., Blazevic V. (2020). Internalization and antigen presentation by mouse dendritic cells of rotavirus VP6 preparations differing in nanostructure. Mol. Immunol..

[18] Peralta A., Molinari P., Taboga O. (2009). Chimeric recombinant rotavirus-like particles as a vehicle for the display of heterologous epitopes. Virol. J..

[19] Teng Y., Zhao B., Pan X., Wen Y., Chen Y. (2014). A new rotavirus VP6-based foreign epitope presenting vector and immunoreactivity of VP4 epitope chimeric proteins. Viral Immunol..

[20] Grohn S., Heinimaki S., Tamminen K., Blazevic V. (2021). Expression of influenza A virus-derived peptides on a rotavirus VP6-based delivery platform. Arch. Virol..

[21] PANGO Lineages. https://cov-lineages.org/.

[22] O’Shaughnessy L., Doyle S. (2011). Purification of proteins from baculovirus-infected insect cells. Methods Mol. Biol..

[23] Lappalainen S., Tamminen K., Vesikari T., Blazevic V. (2013). Comparative immunogenicity in mice of rotavirus VP6 tubular structures and virus-like particles. Hum. Vaccines Immunother..

[24] Cohen A.A., Gnanapragasam P.N.P., Lee Y.E., Hoffman P.R., Ou S., Kakutani L.M., Keeffe J.R., Wu H.J., Howarth M., West A.P. (2021). Mosaic nanoparticles elicit cross-reactive immune responses to zoonotic coronaviruses in mice. Science.

[25] Tan C.W., Chia W.N., Qin X., Liu P., Chen M.I., Tiu C., Hu Z., Chen V.C., Young B.E., Sia W.R. (2020). A SARS-CoV-2 surrogate virus neutralization test based on antibody-mediated blockage of ACE2-spike protein-protein interaction. Nat. Biotechnol..

[26] Tamminen K., Huhti L., Koho T., Lappalainen S., Hytonen V.P., Vesikari T., Blazevic V. (2012). A comparison of immunogenicity of norovirus GII-4 virus-like particles and P-particles. Immunology.

[27] Foged C. (2011). Subunit vaccines of the future: The need for safe, customized and optimized particulate delivery systems. Ther. Deliv..

[28] He Q., Mao Q., An C., Zhang J., Gao F., Bian L., Li C., Liang Z., Xu M., Wang J. (2021). Heterologous prime-boost: Breaking the protective immune response bottleneck of COVID-19 vaccine candidates. Emerg. Microbes Infect..

[29] Lan J., Ge J., Yu J., Shan S., Zhou H., Fan S., Zhang Q., Shi X., Wang Q., Zhang L. (2020). Structure of the SARS-CoV-2 spike receptor-binding domain bound to the ACE2 receptor. Nature.

[30] Overgaard N.H., Frosig T.M., Jakobsen J.T., Buus S., Andersen M.H., Jungersen G. (2017). Low antigen dose formulated in CAF09 adjuvant Favours a cytotoxic T-cell response following intraperitoneal immunization in Gottingen minipigs. Vaccine.

[31] Chan J.F., Zhang A.J., Yuan S., Poon V.K., Chan C.C., Lee A.C., Chan W.M., Fan Z., Tsoi H.W., Wen L. (2020). Simulation of the clinical and pathological manifestations of coronavirus disease 2019 (COVID-19) in a golden Syrian hamster model: Implications for disease pathogenesis and transmissibility. Clin. Infect. Dis..

[32] Zaira R., Ammad F., Faraz B.M. (2021). Scouting the receptor binding domain of COVID-19: A comprehensive immunoinformatics inquisition. Res. Sq..

[33] Lee W.S., Wheatley A.K., Kent S.J., DeKosky B.J. (2020). Antibody-dependent enhancement and SARS-CoV-2 vaccines and therapies. Nat. Microbiol..

[34] Bolles M., Deming D., Long K., Agnihothram S., Whitmore A., Ferris M., Funkhouser W., Gralinski L., Totura A., Heise M. (2011). A double-inactivated severe acute respiratory syndrome coronavirus vaccine provides incomplete protection in mice and induces increased eosinophilic proinflammatory pulmonary response upon challenge. J. Virol..

[35] Tseng C.T., Sbrana E., Iwata-Yoshikawa N., Newman P.C., Garron T., Atmar R.L., Peters C.J., Couch R.B. (2012). Immunization with SARS coronavirus vaccines leads to pulmonary immunopathology on challenge with the SARS virus. PLoS ONE.

[36] Morgan J., Muskat K., Tippalagama R., Sette A., Burel J., Arlehamn C.S.L. (2021). Classical CD4 T cells as the cornerstone of antimycobacterial immunity. Immunol. Rev..

[37] Lee L.Y.H., Simmons C., Jong M.D.D., Chau N.V., Schumacher R., Peng Y.C., McMichael A.J., Farrar J.J., Smith G.L., Townsend A.R. (2008). Memory T cells established by seasonal human influenza A infection cross-react with avian influenza A (H5N1) in healthy individuals. J. Clin. Investig..

[38] DeWitt W.S., Emerson R.O., Lindau P., Vignali M., Snyder T.M., Desmarais C., Sanders C., Utsugi H., Warren E.H., McElrath J. (2015). Dynamics of the cytotoxic T cell response to a model of acute viral infection. J. Virol..

[39] Mothe B., Llano A., Ibarrondo J., Daniels M., Miranda C., Zamarreno J., Bach V., Zuniga R., Perez-Alvarez S., Berger C.T. (2011). Definition of the viral targets of protective HIV-1-specific T cell responses. J. Transl. Med..

